# Different Environmental Drivers of Highly Pathogenic Avian Influenza H5N1 Outbreaks in Poultry and Wild Birds

**DOI:** 10.1371/journal.pone.0053362

**Published:** 2013-01-07

**Authors:** Yali Si, Willem F. de Boer, Peng Gong

**Affiliations:** 1 Ministry of Education Key Laboratory for Earth System Modeling, and Center for Earth System Science, Tsinghua University, Beijing, China; 2 Resource Ecology Group, Wageningen University, Wageningen, The Netherlands; National Institute for Viral Disease Control and Prevention, CDC, China

## Abstract

A large number of highly pathogenic avian influenza (HPAI) H5N1 outbreaks in poultry and wild birds have been reported in Europe since 2005. Distinct spatial patterns in poultry and wild birds suggest that different environmental drivers and potentially different spread mechanisms are operating. However, previous studies found no difference between these two outbreak types when only the effect of physical environmental factors was analysed. The influence of physical and anthropogenic environmental variables and interactions between the two has only been investigated for wild bird outbreaks. We therefore tested the effect of these environmental factors on HPAI H5N1 outbreaks in poultry, and the potential spread mechanism, and discussed how these differ from those observed in wild birds. Logistic regression analyses were used to quantify the relationship between HPAI H5N1 outbreaks in poultry and environmental factors. Poultry outbreaks increased with an increasing human population density combined with close proximity to lakes or wetlands, increased temperatures and reduced precipitation during the cold season. A risk map was generated based on the identified key factors. In wild birds, outbreaks were strongly associated with an increased Normalized Difference Vegetation Index (NDVI) and lower elevation, though they were similarly affected by climatic conditions as poultry outbreaks. This is the first study that analyses the differences in environmental drivers and spread mechanisms between poultry and wild bird outbreaks. Outbreaks in poultry mostly occurred in areas where the location of farms or trade areas overlapped with habitats for wild birds, whereas outbreaks in wild birds were mainly found in areas where food and shelters are available. The different environmental drivers suggest that different spread mechanisms might be involved: HPAI H5N1 spread to poultry via both poultry and wild birds, whereas contact with wild birds alone seems to drive the outbreaks in wild birds.

## Introduction

Highly pathogenic avian influenza (HPAI) H5N1 can spread rapidly over a large geographic area among poultry and wild birds and has also been transmitted from birds to mammals including humans, with high mortality rates [1]. Understanding the environmental drivers of HPAI H5N1 outbreaks is of great importance for identifying high risk areas, setting priorities for preventive actions and developing precautionary measures against future outbreaks. So far, many physical environmental factors (e.g. surface water availability, topography, or climate) and anthropogenic environmental factors (e.g., the distance to roads, poultry density, or human population density) have been associated with HPAI H5N1 outbreaks in poultry and wild birds [2]. However, no analysis has been made in comparing the differences in the underlying mechanisms that drive these outbreak patterns between poultry and wild birds.

Environmental determinants can provide clues to the spread mechanisms underlying the outbreak patterns. Three spread mechanisms are currently under investigation: poultry transport (both commercial and free-ranging poultry), wild bird movement (mainly waterfowl), and poultry-wildfowl interactions. Transport of poultry could be the main cause if the disease pattern is closely related to anthropogenic environmental factors [3]. Wild birds may act as the main spreading agent if the disease outbreak pattern is strongly correlated with physical environmental factors [4]. Virus exchange between poultry and wild birds can be facilitated if poultry trade areas or free-ranging areas are in proximity to wild waterfowl habitats, such as lakes or wetlands [4]–[6]. Hence, the virus could be mainly spread via poultry-wildfowl interactions, with interaction variables (e.g. poultry or human population density combined with the proximity to lakes or wetlands) as key environmental drivers.

**Figure 1:**
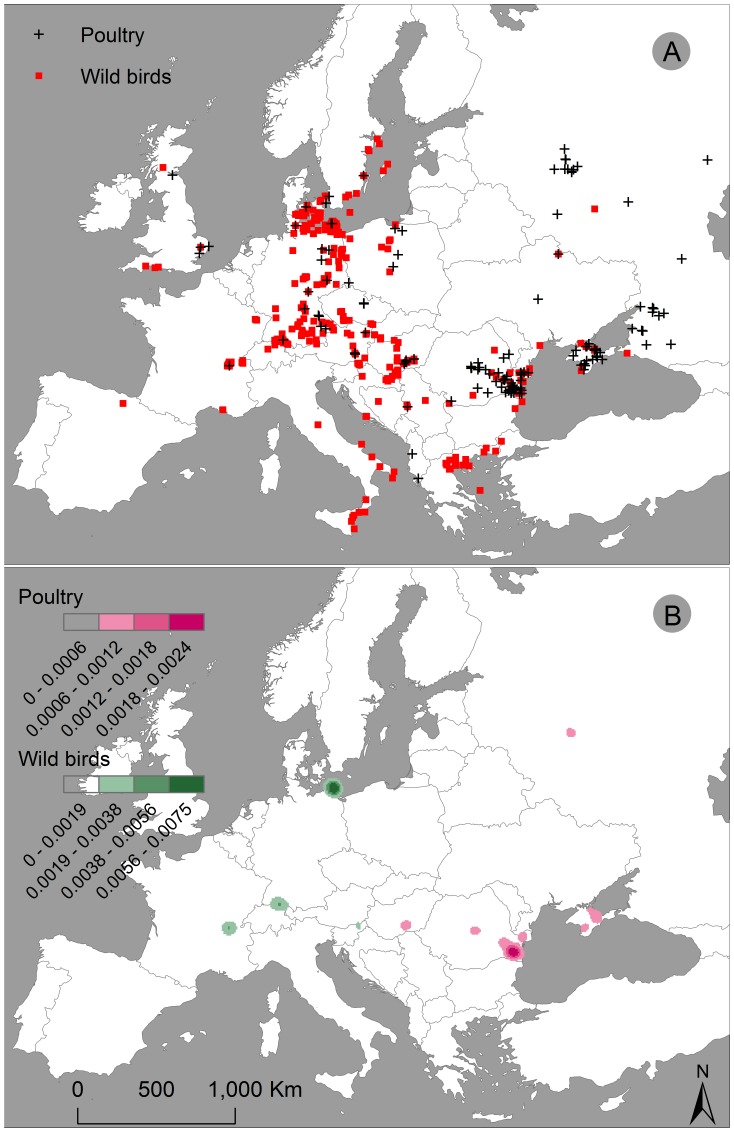
Distribution of confirmed highly pathogenic avian influenza (HPAI) H5N1 outbreaks in Europe from 2005 to 2008. (A) Outbreaks in poultry (red squares) and wild birds (black crosses). (B) Their kernel densities (number of outbreaks/square kilometre).

A large number of HPAI H5N1 outbreaks in both poultry and wild birds have been reported in Europe since 2005. Outbreaks in wild birds are concentrated in the central part of Europe, while outbreaks in poultry are mainly found in the eastern Europe such as the Black Sea region, with sporadic infections mostly around wild bird infections (Fig. 1a). Kernel densities of HPAI H5N1 outbreaks in these two host groups revealed distinct disease outbreak patterns (Fig. 1b). These different spatial patterns indicate that outbreaks in wild birds and in poultry have different environmental drivers, suggesting different spread mechanisms might be involved.

Williams et al. [7] reported no distinct ecological niches of European disease outbreaks in different host groups such as poultry and wild birds. However, their conclusion is only based on the analysis of physical environmental factors (e.g. climate and vegetation indices). The influence of anthropogenic environmental variables and interactions between these the two (e.g. poultry density combined with proximity to wetlands) were not considered.

Si et al. [4] analysed the impact of physical and anthropogenic environmental factors and their interactions on HPAI H5N1 outbreaks in wild birds in Europe. However, the influence of these risk factors in poultry outbreaks has not yet been tested. We therefore investigated the key environmental drivers on HPAI H5N1 outbreaks in poultry, and the potential spread mechanisms, and discussed how these differ from those observed in wild birds.

## Materials and Methods

### Data Collection and Management

The time and locations of 170 confirmed events of HPAI H5N1 outbreaks in poultry in Europe, reported from 2005 to 2008, were acquired from the Global Animal Disease Information System EMPRES-I (http://empres-i.fao.org/eipws3g/) provided by FAO. Three groups of environmental variables were complied, corresponding with different spread mechanisms: anthropogenic environmental (poultry-dominant), physical environmental (wildfowl-dominant), and (poultry-wildfowl) interaction variables.

The anthropogenic environmental variables include human population density in 2005, poultry density in 2005, and distance to the nearest city, metropolis, road, highway, and railway. The physical environmental variables comprise distance to the nearest lake or wetland, distance to the nearest Ramsar site, digital elevation model (DEM) and derived slope and aspect, mean annual potential evapotranspiration, mean annual aridity index, mean monthly precipitation, mean monthly minimum and maximum temperature, and monthly Normalized Difference Vegetation Index (NDVI). Monthly data were included to consider potential seasonal effects. Four 12-month NDVI data sets from 2005 to 2008 were smoothed by employing an adaptive Savitzky-Golay smoothing filter, using the TIMESAT package [8]. The 12-month NDVI time series used in this study were reconstructed by averaging these 4 (2005–2008) smoothed NDVI series. A close proximity to lakes, wetlands or Ramsar sites indicates a higher chance of the presence of wild birds, which is expected to be positively correlated to disease outbreaks. Distance to the nearest lake or wetland and distance to the nearest Ramsar site were then converted to proximity to lakes or wetlands and to Ramsar sites to match the potential positive effect of poultry and human population density on disease outbreaks. Four interaction variables were constructed by multiplying proximity to lakes or wetlands and proximity to Ramsar sites with poultry density and human population density. These interaction variables reflect environmental conditions where poultry and wild birds potentially meet, which is expected to increase contact opportunities and disease risk. Table 1 summarizes the name, abbreviation, unit, and data source of the environmental variables used in this study. Detailed links of data sources are provided in Table S1. The potential associations between these environmental factors and HPAI H5N1 outbreak patterns have been demonstrated by previous studies [2]. We are particularly interested in testing their interaction effects and identifying the key environmental factors that most strongly influence HPAI H5N1 outbreak patterns.

**Table 1:** Summary of the anthropogenic, physical environmental variables and interaction variables used in the analysis.

Category	Description of variables	Abbreviation	Unit	Data source
Anthropogenic	Distance to the nearest city	City	km	ESRI
environmental	Distance to the nearest metropolis	Metro	km	ESRI
variables	Distance to the nearest road	Road	km	ESRI
	Distance to the nearest highway	Highway	km	ESRI
	Distance to the nearest railway	Railway	km	ESRI
	Human population density in 2005	Hpopden	p/km^2^	CIESIN, FAO, CIAT
	Poultry density in 2005	Poultryden	p/km^2^	FAO
Physical	Distance to the nearest lake or wetland	GLWD	km	WWF, ESRI, CESR
Environmental	Distance to the nearest Ramsar site	Ramsar	km	Wetlands International
variables	Digital elevation model	DEM	m	CGIAR-CSI
	Slope aspect	Aspect	°	–
	Slope gradient	Slope	°	–
	Mean annual potential evapotranspiration	Mapet	mm/km^2^/year	CGIAR-CSI
	Mean annual aridity index	Maaridity	No unit	CGIAR-CSI
	Mean monthly precipitation	PrecJan to Dec	mm	WORLDCLIM
	Mean monthly minimum temperature	TminJan to Dec	°C*10	WORLDCLIM
	Mean monthly maximum temperature	TmaxJan to Dec	°C*10	WORLDCLIM
	Monthly NDVI	NDVIJan to Dec	No unit	NASA
Interaction	Human density * proximity to lakes or wetlands	Popdenglwd	No unit	–
variables	Human density * proximity to Ramsar sites	Popdenram	No unit	–
	Poultry density * proximity to lakes or wetlands	Poultrydenglwd	No unit	–
	Poultry density * proximity to Ramsar sites	Poultrydenram	No unit	–

Localities where HPAI H5N1 outbreaks had been reported before tend to be more intensively surveyed/sampled than localities where no outbreaks were reported. Duplicated outbreaks of HPAI H5N1 from the same locality were discarded to reduce this sampling bias, resulting in 133 unique geographic coordinates. A disease presence area was constructed by generating 10 km radius buffers around each presence location, as Europe adopted a 10 km surveillance zone policy [9]. The disease absence area was defined as the area within the minimum convex polygon of all poultry outbreaks in Europe, excluding the presence area. A total of 5000 absence locations were generated randomly in this absence area. The minimum distance between locations was set at 20 km to avoid overlapping surveillance buffers. Values were extracted from the 7 distance layers for all presence and absence locations: distance to the nearest city, metropolis, road, highway, railway, lake or wetland, and Ramsar site. The mean values within a 10 km buffer zone instead of a single extracted value was calculated for each location to represent the environmental conditions of the remaining layers: human population density, poultry density, elevation, slope aspect, slope gradient, potential evapotranspiration, aridity index, precipitation, minimum temperature, maximum temperature, NDVI, and interaction variables.

### Statistical Analysis

**Figure 2:**
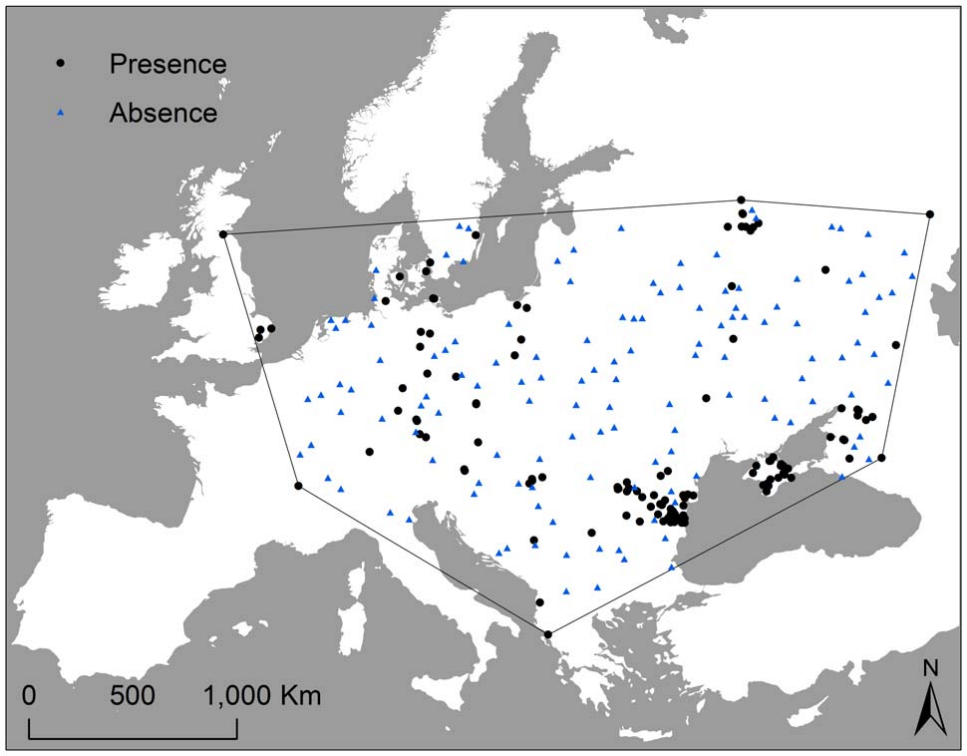
Distribution of presence and absence of highly pathogenic avian influenza (HPAI) H5N1 occurrences in poultry in Europe from 2005 to 2008. The polygon indicates the minimum convex polygon of poultry infections. Black dots indicate presence and blue triangles indicate absence (one training subset).

Logistic regression analyses were utilized to examine the relationship between explanatory variables and the HPAI H5N1 outbreaks in poultry. A bootstrapping procedure was adopted in which, together with the 133 presence locations, 133 absence locations were randomly selected with replacement from the 5000 absence locations. This process was repeated 1000 times, creating 1000 subsets for model training (see Fig. 2 for an example of one training subset).

Univariate logistic regression analyses were firstly applied to investigate both linear and quadratic effects of each variable on the outbreak of HPAI H5N1 in poultry. The relationship was evaluated by the mean odds ratios (OR), 95% confidence intervals (CIs) of OR, the *P*-value, Akaike’s information criterion (AIC), and the area under the Receiver Operating Characteristic curve (AUC) calculated from the 1000 training subsets. Variables with *P*-values ≥0.1 were excluded from further analyses. Variables with relatively high collinearity or high spatial autocorrelation were dropped by examining the variance inflation factors (VIF) and Moran’s I, respectively. Variables were removed by sequentially dropping the variable with the lowest impact, recalculating the VIFs and repeating this process until all VIFs were <10 [10]. Variables with a Moran’s I ≥0.5 or ≤-0.5 were also removed.

Multiple backward stepwise logistic regression was carried out to select the significant independent variables. This stepwise process was repeated 1000 times using the different training subsets. The frequency of each variable being selected was calculated on the basis of applying 1000 best stepwise logistic regression models, ranked by AIC. A mean *P*-value was calculated for each selected variable, and variables yielding non-significant effects (mean *P*-value >0.05) were discarded.

Multiple logistic regression was then carried out using the remaining variables. This process was repeated 1000 times using the different training subsets. The mean values of coefficients, OR, 95% CIs of OR, *P*-value, AIC, and AUC were used as indicators of model performance. Only significant variables (*P*-value ≤0.05) were retained in the final model.

All variables were standardized (z-score) prior to the analyses. All statistical analyses were conducted using R statistical software (www.r-project.org). R codes are available in Appendix S1 in Supporting Information.

The risk map was generated based on the multiple logistic regression model defined as: 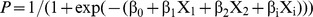



where *P* is the probability of disease outbreaks, β_0_ is the constant, X_1_,…X_i_ are the key environmental factors, and β_1_,…β_i_ are their regression coefficients.

## Results

Positive linear relationships were found between poultry outbreaks and population density, temperature (minimum and maximum), and several interaction variables (human population density combined with the proximity to lakes or wetlands and Ramsar sites, poultry density combined with the proximity to lakes or wetlands). Negative linear associations were recorded between poultry outbreaks and DEM, slope, potential evapo-transpiration, precipitation and NDVI (Apr to Sep). Precipitation and maximum temperature (Sep-Nov) had significant positive quadratic terms (upward parabola) while maximum temperature (Mar) and NDVI (Jan) showed significant negative quadratic terms (downward parabola).

Seven variables were kept after the stepwise selection (mean *P*-value ≤0.05) (Table 2). The minimum temperature (Apr), the maximum temperature (Nov), NDVI (Jan) and human population density combined with the proximity to lakes or wetlands all showed positive effects. Precipitation (Apr, Sep and Nov) showed negative effects. Significant positive quadratic terms were observed for precipitation (Nov) and maximum temperature (Nov), and a significant negative quadratic term was found for NDVI (Jan).

**Table 2:** Environmental variables kept after the process of stepwise selection using 1000 bootstrapping training datasets. Italics indicate quadratic effects.

Univariate and quadratic logistic regression								Stepwise selection		
Variable	OR	95% CIs OR		*P-*value	AIC	AUC	Moran’s I	VIF	Times	Mean *P*-value
PreApr	0.963	0.942	0.985	0.008	350	0.62	0.21	7.83	540	0.035
PreSep	0.956	0.937	0.975	<0.001	340	0.69	0.30	6.29	696	0.026
*PreNov*	*0.956*	*0.943*	*0.969*	*<0.001*	*773*	*0.66*	*0.15*	*4.06*	*904*	*0.009*
*SpreNov*	*1.231*	*1.123*	*1.350*	*0.010*					*694*	*0.040*
TminApr	1.041	1.026	1.056	<0.001	329	0.70	0.24	4.70	246	0.050
*TmaxNov*	*1.023*	*1.017*	*1.029*	*<0.001*	*724*	*0.75*	*0.40*	*6.87*	*556*	*0.026*
*STmaxNov*	*1.366*	*1.163*	*1.603*	*0.020*					*967*	*<0.001*
*NDVIJan*	*20.869*	*3.671*	*119.904*	*0.026*	*805*	*0.60*	*0.25*	*1.86*	*769*	*0.036*
*SNDVIJan*	*0.739*	*0.629*	*0.868*	*0.007*					*634*	*0.049*
popdenglwd	1.070	1.011	1.132	0.076	355	0.63	0.01	1.13	982	0.016

OR - odds ratios, 95% CIs OR −95% confidence intervals of odds ratios, AIC - Akaike’s information criterion, AUC - the area under the Receiver Operating Characteristic curve, VIF - variance inflation factor, Prec - precipitation, Tmin - minimum temperature, Tmax - maximum temperature, S - square term, Popdenglwd - human population density combined with the proximity to lakes or wetlands.

**Figure 3:**
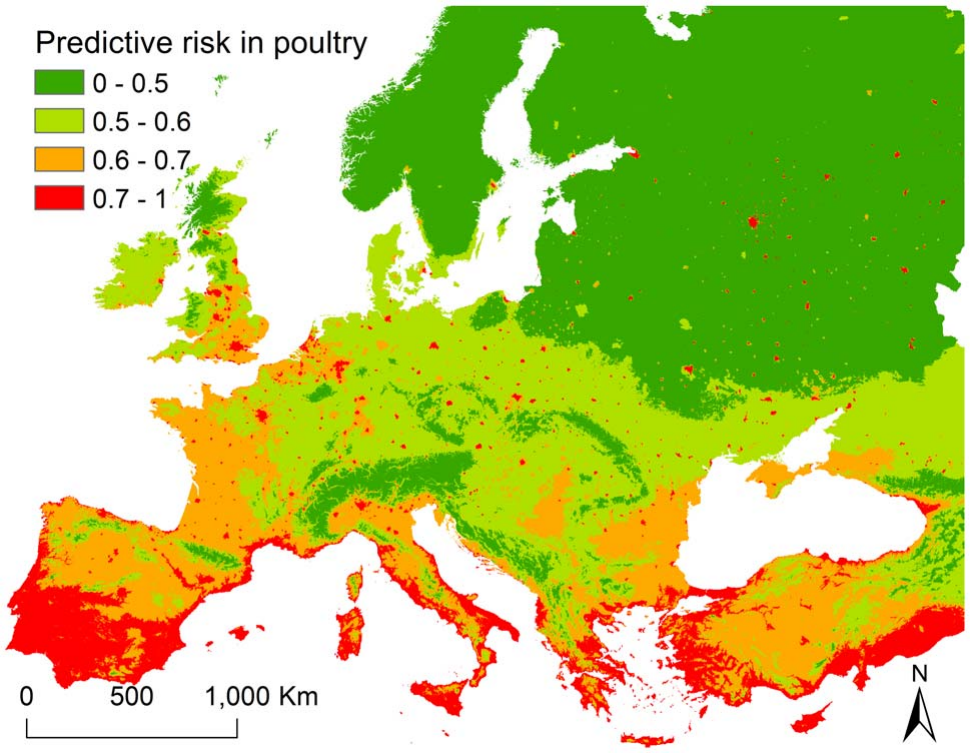
Predictive risk map of highly pathogenic avian influenza (HPAI) H5N1 outbreaks in poultry in Europe.

In the final analysis, three variables were identified as key risk factors strongly influencing HPAI H5N1 occurrence in poultry in Europe. Reduced precipitation (Nov), increased maximum temperatures (Nov) and an increased human population density combined with close proximity to lakes or wetlands all increased the probability of HPAI H5N1 outbreaks in poultry. A predictive risk map of HPAI H5N1 outbreaks in poultry was generated based on the final multiple logistic regression analysis (Fig. 3). High-risk areas were mainly located in the Black sea region, the western and the southern part of Europe. A large number of scattered high-risk spots was observed across the central and eastern part of Europe.

Table 3 shows the key environmental drivers of HPAI H5N1 outbreaks in poultry (this study) and compared these to those observed in wild birds [4]. All significant factors influencing wild bird infections were physical environmental variables, while the key factors affecting poultry infections include both physical environmental and interaction variables. The probability of both outbreak types increased with increasing temperatures and reduced precipitation during the cold season. However, poultry infections were significantly associated with a higher human population density combined with the close proximity to lakes or wetlands, while wild bird infections were strongly correlated to increased NDVI and lower elevations.

**Table 3:** Comparison of significant environmental factors correlated to HPAI H5N1 outbreaks in poultry (this study) and wild birds (adapted from [4]) in Europe, as maintained in the final multiple logistic regression model.

Type	Poultry	B	OR	95% CIs OR		*P*-value	AIC ± SD	AUC ± SD
	Model					<0.001	283±18	0.81±0.03
	Intercept	−0.16				0.462		
Physical	PrecNov	−0.05	0.955	0.936	0.973	<0.001		
environmental	TmaxNov	0.03	1.027	1.017	1.037	<0.001		
factors	STmaxNov	0.45	1.586	1.228	2.049	0.025		
interaction	Popdenglwd	0.26	1.319	1.08	1.629	0.042		
	**Wild birds**							
	Model					<0.001	638±25	0.81±0.02
	Intercept	−10.9				<0.001		
Physical	Dem	−0	0.999	0.998	0.999	0.016		
environmental	PrecJan	−0.04	0.963	0.950	0.976	<0.001		
factors	TminJan	0.03	1.026	1.018	1.034	<0.001		
	NDVIMar	50.6	1.170	0.059	23.630	<0.001		
	SNDVIMar	−56.4	0.408	0.307	0.543	<0.001		
	NDVIDec	6.1	1319	100	17685	<0.001		

OR - odds ratios, 95% CIs OR −95% confidence intervals of odds ratios, AIC - Akaike’s information criterion, AUC - the area under the Receiver Operating Characteristic curve, Prec - precipitation, Tmax - maximum temperature, Tmin - minimum temperature, S - square term, Popdenglwd - human population density combined with the proximity to lakes or wetlands.

## Discussion

Different environmental drivers operate on HPAI H5N1 outbreaks in poultry and wild birds in Europe. The probability of HPAI H5N1 outbreaks in poultry increases in areas with a higher human population density and a shorter distance to lakes or wetlands. This reflects areas where the location of farms or trade areas and habitats for wild birds overlap. In wild birds, HPAI H5N1 outbreaks mostly occurred in areas with increased NDVI and lower elevations which are typically areas where food and shelter for wild birds are available [4]. The association with migratory flyways has also been found in the intra-continental spread of the low pathogenic avian influenza virus in North American wild birds [11]. These different environmental drivers suggest that different spread mechanisms operate. Disease might spread to poultry via both poultry and wild birds, through direct (via other birds) or indirect (e.g. via contaminated environment) infection. Outbreaks in wild birds are mainly caused by transmission via wild birds alone, through sharing foraging areas or shelters [4].

These findings are in contrast with a previous study [7], which did not find environmental differences between disease outbreaks in poultry and wild birds in Europe. The influence of physical environmental factors on outbreaks in poultry and wild birds is indeed similar as the outbreak probability increases with increasing temperatures and reduced precipitation during the cold seasons. However, the influence of anthropogenic environmental factors and interaction factors is different, which could explain the different spatial patterns observed in HPAI H5N1 outbreaks between poultry and wild birds (Fig. 1).

Human population density combined with the proximity to lakes or wetlands consistently showed a positive relationship with HPAI H5N1 outbreaks in poultry. Both human population density and the presence of water were identified as one of the most common environmental factors influencing HPAI H5N1 outbreaks across different regions and spatial scales [2]. A higher human population density reflects large amount of poultry production or trade activities, resulting in an increased risk of contact with infected poultry. Poultry production activities or trade in proximity to wetlands or lakes would increase the chances of infection because of the higher risk of contact with infected domestic waterfowl, infected wild birds, or contaminated environment. The HPAI H5N1 virus can survive in water or bird faeces for extended periods, especially at low temperatures as water remains infectious for up to 207 days at 17°C or up to 102 days at 28°C [12] and the HPAI H5N1 virus remains virulent in liquid bird faeces for 30–35 days at 4°C and for 7 days at 20°C [13]. The frequent reoccurrences of disease clusters in the Black Seas region could be caused by viruses surviving in contaminated water or bird faeces in the environment [6]. Similar to our findings, Ward et al. [5] also found that HPAI H5N1 outbreaks in village poultry populations in Romania were significantly associated with villages situated less than 5 km from a river or stream. The distribution of human population combined with the close proximity to rivers or wetlands is an important interaction gate between poultry and wild birds.

This study emphasizes the substantial contribution of climatic factors, in particular, temperature and precipitation, on HPAI H5N1 outbreaks in wild birds and poultry. The association between poultry infections and climatic factors could be explained by the direct or indirect contact (e.g. through contaminated environment) with wild birds. The link between disease outbreaks in wild birds and climatic factors has been reported by previous studies. For instance, Ottaviani et al. [14] and Reperant et al. [15] demonstrated that spatio-temporal patterns of HPAI H5N1 outbreaks in wild birds in Europe were associated with regions south of the 0°C isotherm, i.e. areas that are relatively warmer during the winter and where wild birds aggregate. Farnsworth et al. [16] also suggested that viral deposition in water and sub-freezing temperatures act as determinants of avian influenza infection in wild waterfowl across North America. Besides of bird aggregation, an increased temperature can also stimulate viral activity, which explains the positive linear effect reported in the univarate and stepwise analyses. Maximum temperature in November consistently showed a significant positive quadratic term, indicating a negative relationship at the low temperature range, probably due to the extended survival time of the virus in the environment under low temperatures. Reduced precipitation or low evapo-transpiration may lead to reduced foraging areas, which increases local aggregation of both free-ranging domestic and wild bird populations.

Some physical environmental factors, such as elevation and NDVI, consistently correlated with the outbreak patterns of wild birds, but were dropped from the final risk model of poultry infections (Table 3). These two factors are closely related to wild bird distribution and movement. For example, lower elevation and slope are found in lowlands or floodplains, potentially important waterfowl habitats [3]. Areas with lower/intermediate NDVI (small plants such as grasses and herbaceous plants) are more attractive to waterfowl than those of higher NDVI (typical found in forests) [4]. Hence, the negative/quadratic effects that elevation and NDVI had on poultry infections suggest that wild birds are involved in the spread of HPAI H5N1 to poultry.

The level of bio-security, preventive measures designed to reduce the risk of disease transmission, may negatively influence the sensitivity of the model to environmental factors. Poultry infections would be decreased if poultry are quarantined from contact with other birds. In this case, areas with a higher poultry density might not necessary have a higher risk. Hence, in line with previous findings in China [17], [18], we also found that an increased poultry density did not lead to an increased risk of HPAI H5N1 outbreak. The intensive commercial poultry production systems in Europe may have a relatively high level of bio-security, in contrast to some regions where poultry are free ranging during daylight hours, such as the free-grazing poultry areas in the Danube River delta [5].

We suggest that areas with a relatively high human population density and also close to wetlands or lakes, facilitated by increased temperatures and reduced precipitation during the cold seasons (Fig. 3) should be the target of early detection of HPAI H5N1 outbreaks in poultry. Improving the bio-security level at these areas should be a priority to reduce future HPAI H5N1 outbreaks.

## Supporting Information

Table S1 **Summary of the links to the data sets used in this study.**
(DOCX)

Appendix S1 **R scripts used to perform the analysis.**
(R) Click here for additional data file.
