# Supplementary material for: Different Environmental Drivers of Highly Pathogenic Avian Influenza H5N1 Outbreaks in Poultry and Wild Birds
Source: PLoS One. 2013 Jan 7;8(1):e53362. doi: 10.1371/journal.pone.0053362 (PMC3538778; doi:10.1371/journal.pone.0053362)
Supplement: Table S1 — Summary of the links to the data sets used in this study. (DOCX) [file pone.0053362.s001.docx]

Table S1. Summary of the links to the data sets used in this study.

| **Description of data sets** | **Links** |
| --- | --- |
| Cities | http://www.esri.com/data/data-maps/ |
| Metropolises | http://www.esri.com/data/data-maps/ |
| Roads | http://www.esri.com/data/data-maps/ |
| Highways | http://www.esri.com/data/data-maps/ |
| Railways | http://www.esri.com/data/data-maps/ |
| Human population density in 2005 | http://sedac.ciesin.columbia.edu/data/set/gpw-v3-population-density-future-estimates |
| Poultry density in 2005 | http://www.fao.org/geonetwork/srv/en/metadata.show?id=12720 |
| Global lakes and wetlands database | http://worldwildlife.org/publications/global-lakes-and-wetlands-database-lakes-and-wetlands-grid-level-3 |
| Ramsar sites | http://ramsar.wetlands.org/GISMaps/DownloadGISdatasets/tabid/769/Default.aspx |
| Digital elevation model | http://srtm.csi.cgiar.org/SELECTION/inputCoord.asp |
| Mean annual potential evapotranspiration | http://www.cgiar-csi.org/2010/04/134/ |
| Mean annual aridity index | http://www.cgiar-csi.org/2010/04/134/ |
| Mean monthly precipitation | http://www.worldclim.org/current |
| Mean monthly minimum temperature | http://www.worldclim.org/current |
| Mean monthly maximum temperature | http://www.worldclim.org/current |
| Monthly NDVI | ftp://e4ftl01.cr.usgs.gov/MODIS_Composites/MOLT/MOD13A3.005/ |
